# Hering Memorial Hospital

**Published:** 1887-11

**Authors:** 


### THE HERING MEMORIAL HOSPITAL.

OPENING OF THE HANDSOME HOSPITAL OF THE WOMEN'S HOMŒOPATHIC ASSOCIATION AT TWENTIETH STREET AND SUSQUEHANNA AVENUE—A GREAT THRONG OF PEOPLE ATTEND THE CEREMONIES.

The magnificent edifice just erected at Twentieth Street and Susquehanna Avenue by the Women's Homœopathic Association of Pennsylvania, and forming one of a series of hospital buildings to be erected under the auspices of that organization, was formally opened October 13th, in the Board room of the new building, in the presence of a great throng of eminent men and women of the homœopathic school. Distinguished citizens in other professional walks and many others interested in the important movement crowded the audience room to overflowing.

What a grand epoch would the opening day of this hospital building have been to Constantine Hering had he lived to see it! That great master mind, who lived, labored (we may almost say loved) only for pure Homœopathy, would indeed have rejoiced to have seen this hospital, probably the only one in the world devoted solely to homœopathic practice.

This building is most appropriately called the Hering Building, for there medicine *is* practiced as Hering taught, practiced, and believed. While this hospital is a fitting memorial to Hering, it is also a monument to woman's energy, perseverance, and charity. Laboring under the greatest difficulties, opposed by the vast majority of Philadelphia physicians (for the allopath and the eclectic homœopath united in opposing a hospital erected for the practice of *pure* Homœopathy), the ladies of the Women's Homœopathic Association of Pennsylvania have achieved a great success, which should receive the warmest acknowledgment homœopaths can make. The ladies instrumental in bringing it about should receive the highest honor in our power to bestow. Their individual names should be familiar in our minds as those of any benefactors of mankind.

The ceremonies incident to the installation of the noble work were presided over by Mrs. Mary W. Coggins, President of the Institution, who introduced Rev. Dr. H. L. Wayland, who invoked the Divine blessing upon those who, under His guidance, had completed a work that would be a harbinger for good in the ages to come. He asked for spiritual aid for the physicians, the matron, and the nurses in the discharge of their trying and oft-times repugnant work.

Dr. Lippe then delivered the opening address.

Ladies and Gentlemen: We have met here to-day to celebrate the opening of the medical, surgical, and maternity hospitals established by the Women's Homœopathic Association of Pennsylvania. The Association has honored me with an invitation to deliver the opening address on this occasion, and permit me to ask your forbearance with my humble attempt to do full justice to the task before me. The hospital now opened is the result of unceasing efforts to extend the blessings of Homœopathy to the variously afflicted sick, and especially to *women* in need of medical care.

The Women's Homœopathic Association have taken one more step forward to extend the healing art promulgated by the immortal sage and philosopher, Samuel Hahnemann, and to demonstrate its blessings practically and publicly in this hospital. Philadelphia may feel a just pride this day at the achievement of this noble Association. This city was the cradle of Homœopathy; in this city that infant was nursed by the late Dr. Constantine Hering; and we see here before us a practical illustration of the successful nursing of it; we see here before us a new generation of benevolent women, who in a substantial manner will nurse again the child of Samuel Hahnemann, his method of applying the healing art under the operation of the only natural law governing the medical art—the law of similars. The early pioneers of the homœopathic healing art were able to demonstrate the great success in healing the sick in comparatively isolated cases, and this day we welcome the opening of a magnificent hospital in which it will be better possible to demonstrate the progressive success of homœopathic treatment—progressive under all aids which hygiene and perfect nursing can give otherwise appropriate and skillful medical treatment. Isolated cases of the success of homœopathic treatment have done much to bring credit to our healing art, but when we shall demonstrate how collectively the results will become convincingly greater than could appear by the examples of isolated cases, we must highly appreciate the good influence emanating from this hospital. Especially true is this when we contemplate what figures will and must show of the benign results of pure homœopathic treatment on the large scale; how suffering can be diminished to a minimum without the pernicious use of Chloroform, Ether, Ergot, and all the daily palliatives and local treatments resorted to by the old school of medicine. The hospital will show in its reports the great superiority of the homœopathic healing art over other curative methods; and under the efficient nursing of this Association of charitable and earnest ladies, this hospital is sure to do good service in advancing the healing art.

Let us look for a moment into the past before we turn to look more into the future. Philadelphia is a fortunate city; Divine Providence has favored it in a remarkable manner. From this city emanated the Declaration of Independence, and we have just celebrated the centennial anniversary of the adoption of the Constitution of the United States. Living, as we do, under the protection of this glorious Constitution, we have witnessed our healing art progressing prosperously in a measure impossible for it under other forms of government, where oppressive laws favor the old school of medicine, and where our healing art meets with all kinds of hindrances to its advancement. In this cradle of true liberty, liberty in medical matters was also nursed along with political liberty, liberty of conscience and religion, liberty of the press, and liberty of speech. Medical tyranny is not to be thought of in this land where true liberty is appreciated and sustained by the will of the people. All opposition by misguided opponents to our healing art and to the fullest liberty in medicine will be as fruitless as would be the efforts of misguided opponents of our glorious Constitution. Philadelphia, the cradle of universal liberty, protected by the Constitution, is also become the cradle of Homœopathy. For, to me, there is a great analogy between them. Our Constitution is based on the assumption that human rights, including personal liberty, have their origin in natural and Divine laws existing since the creation of the world. Divine Providence guided the framers of the Constitution in applying these laws practically for the good of the people. Homœopathy is based also on a natural and Divine law existing since the creation of the world. The correctness of our law, the law of the similars, has never been doubted, but it was left to Divine Providence to inspire and direct Samuel Hahnemann to apply this law practically for the good of the sick.

What may we reasonably expect from the future? A hospital that, under the kind care of the Women's Homœopathic Association, shall be the means of restoring to health the sick and the suffering, and by its necessarily superior results further the more universal adoption of our healing art; that shall also offer an opportunity for practical instruction in the homœopathic healing art to the student of medicine who desires to become acquainted with it. The successes of our methods will gain the praises and thanks of the persons benefited and their friends. Physicians of this hospital who do the work will prevent them from falling into the hands of anti-homœopathists. They will in future always seek to obtain such treatment as benefited them. Every person leaving this hospital practically convinced of the truth of the philosophy of Homœopathy will help to further our cause. We live in an age of progress. As in the last half of a century the sailing vessel has been superseded by the steamer, communication by letters has been superseded by the telegraph and the ocean cable, and so also will the antiquated school of medicine have to give way to progressive Homœopathy. Those who seek material causes of disease aided by the microscope will seek in vain, and be compelled at last to accept our knowledge of the dynamic causes of disease. Their material attempts to annihilate the germs of disease by the aid of crude drugs will have to pass into oblivion, and dynamized remedies will be used instead. The hospital this day opened will materially aid in the good progress in expectation. Thanks have to be offered to the noble women of this Association for the good work so assiduously performed in securing the benevolent material means given them, as well as the painstaking, self-sacrificing, charitable work of the medical staff and their corps of nurses; the community at large is beholden to them. And now permit me to add my thanks in the name of all the homœopathic physicians who, in this country as well as the whole world where the homœpathic healing art is practiced, must be gladdened by the celebration of this day.

Dr. J. T. Kent, of St. Louis, should have been the next speaker according to the programme, but he was prevented by urgent professional engagements from appearing, to the great disappointment of the audience, many of whom had come especially to hear him. In his absence it was anticipated that the draft of his address would be on hand to be read by proxy, but it failed to arrive in time.

Dr. Alice B. Campbell, of Brooklyn, was the next speaker.

### ADDRESS OF DR. ALICE B. CAMPBELL.

Ladies and Gentlemen: It gives me more pleasure than I can express to be present with you at this time, and to be identified with this occasion.

The ceremonies of to-day can excite only feelings of gladness in the hearts of all lovers of the sublime law of Homœopathy, and of all admirers of its giant representative, Constantine Hering.

But these ceremonies have a deeper significance and excite a deeper interest than could be aroused by the mere dedicating of a building to hospital purposes. Though this be worthy of notice and praise, they mean more than the designating a hospital department by commemorating the name of one loved and great in the profession of medicine, and worthy of all honor. These acts are commendable and desirable, but they are not the outcome of a necessity. For your city, like most great cities, suffers no lack in hospital facilities. The name of Hering does not depend upon these walls to perpetuate its memory. For, indelible in literature, glorified as scientist and philosopher, emblazoned in the history of Homœopathy, and enshrined in the hearts of its practitioners, what more could any name require to render it immortal.

This occasion gives us joy because it occurs in the interests of truth and uprightness. Truth, as opposed to ignorance and falsehood; uprightness, as set against cowardice and dishonesty. Long enough has the spurious been passing for the genuine. Too long has public confidence been misled and the community deceived. And now, through the stand which the management of this hospital has taken, and which this event celebrates, you cause us to hope that the cloud is to be lifted from the hearts of those who have long deplored a state of things they were powerless to change, for Hahnemannian Homœopathy is to be the rule of practice in this institution. The unmixed and pure law is to have full sway. The weights of stupidity and duplicity, that have so often and so long hindered the soaring aloft of this wonderful truth, have been cut away. Order has supplanted confusion, and light has routed darkness.

Is not the prospect cheering, when we remember this hospital, committed to a system of therapeutics resting upon a sound basis, will produce results commensurate with the perfection of its underlying law? Is it not gratifying to feel assured that the unfortunate and suffering of our kind will here receive at the hands of its ministers such truly scientific and beneficent treatment as will give them the surest relief from pain and the most rapid return to health? For we recognize in Homœopathy a gospel of love to sick humanity, and—pardon a private belief—they will the best execute this mission whose nature and heart are surcharged with this element.

We know that, numerically, Homœopathy is in the minority, but you, by this demonstration, have strengthened our faith in the soundness of that sentiment of Alexander Dumas, when he writes that "majorities prove only what is; minorities are the germ of what should be and what will be."

Else, what means this list of twenty-three names at the head of this Institution, and at the head of this right-about-face movement. None of them belong to the medical profession, and they all belong to—I am proud to say—women. It means that the laity are acquiring correct ideas of Homœopathy; that a knowledge of the effectiveness of a practice according with law is no longer limited to the few in the medical profession. It means that intelligence on this subject is making headway among the people, and that those who use Homœopathy as a business are not the only ones to perceive the beauty of its operations and to appreciate its grand results. It means that the brains of the community have discovered the gauze of the homœopathist, and will no longer tolerate the seeming for the real.

The history of this hospital sustains these assertions—they have had the false, now they will have only the true—and it is pleasant to be able to add, that throughout the land in many homes the echo to these notes is resounding louder every year. It is the quality of such steadily accumulating testimony which confirms the hope that Hahnemannian Homœopathy will eventually occupy an undisputed majority.

Ladies of the "Women's Homœopathic Association of Pennsylvania," you are to be congratulated on the progress you have made toward your ultimate design, as you give token of to-day. We rejoice with you on this added triumph to your labors, and shall continue our sympathy with you in your efforts to complete this hospital and to conduct its wards on the only known law of cure.

The stand which you have taken in demanding homœopathic treatment in a homœopathic hospital is the result of knowledge and of conviction. With such conviction you could do nothing less than make rigid exactions. For law admits of no compromise. You only required of others that which your consciences required of you. In this you have won the confidence of all sincere people, and shown yourselves eminently fitted to command the Institution which you represent, and to care for the interests confided to your keeping.

You have shown courage in doing now that which older institutions should have done before, and in thus doing have set an example worthy of emulation by many other like institutions throughout this broad land, and flying the same professed flag.

It is not your fault that the move you made partook of the radical element, but rather to your credit that you defined and accepted the situation.

Women do not crave leadership, but when duty urges, and there is no one for them to follow, they are not to cringe if in a forward step they are urged against the lever which moves the world.

Progress is not in the hand of either sex, but it is a noticeable fact that the moral element in a momentous question—without the moral element no question can be momentous—is to woman the *reveille* which rouses her from her native retiredness and forces her into the field of action.

Ladies, the road you have marked out for yourselves is not a smooth one, but remember, all progress is rated by the obstacles overthrown, and the main difficulty in any enterprise is removed after a start is effected. You have that start, and now there can no obstruction arise on your way to the topmost achievement that may not be leveled by your hand so long as you continue to act from the standpoint which you now occupy.

Such an institution as this is one of which only an advanced civilization can boast. Created and sustained by women for the sick of her kind, here she can care for her sex as only her sex can care for each other. With trained women nurses, women resident physicians, women doctors on the medical staff, sick woman will be spared many humiliations consequent upon the peculiarities of her ailments. But better than all this—though this is better than could be obtained anywhere a score of years ago—with a medical corps whose intelligence and experience raises them above the need of mechanical treatment of disease and enables them to rely solely on internal medication in all non-surgical cases, there opens up to the patients in this hospital a safe, pleasant, and uncommon road to health, one divested of barbarities and stupid interferences, so prevalent elsewhere, and which are a disgrace to the age.

In your platform of principles you have taken a wide departure from many popular theories and modern innovations, the result of which has been the ousting of crude drugs and the sending of Quinine and Morphine spinning out of doors. This savors largely of a reform in hospital practice, one long needed, and which you are not bringing about any too soon. That a reform may meet with adequate results, the spirit of the martyr must possess the reformer. Ladies, the eyes of the entire homœopathic profession will be henceforth upon this Hering building. Let not the name of this grand and noble genius be unworthily associated. Therefore stand fast in the faith of that cause whereunto you were called. So shall you reap if you swerve not, and your labors shall not return unto you void.

A telegram of congratulation was then read from the veteran Dr. E. A. Ballard, of Chicago, the well-known and efficient Secretary of the International Hahnemannian Association, after which Dr. Wesselhœft delivered the following powerful address:

### ADDRESS OF DR. WILLIAM P. WESSELHŒFT.

Ladies and Gentlemen: Every disciple of Hahnemann in this country, aye, in this world, should to-day rejoice with us in the extraordinary event of the opening of a hospital consecrated to Homœopathy. But why is it so extraordinary an event? Many will say that there are already in this country scores of homœopathic hospitals and dispensaries, a number of which have been in active operation for nearly a score of years. Why then should this new institution merit such esteem and send a thrill of joy through the heart of every homœopathist in the land?

I answer, because it is the first and only hospital in this country, if not in the world, in which no other practice is tolerated but Homœopathy pure and simple. No experiments but those strictly in accord with the system of therapeutics founded by Hahnemann can be allowed in these halls now and forevermore. This, and this alone, should be the cause for rejoicing. We may admire the architectural beauty of the elevation, the admirable arrangements for light, air, ventilation, and comfort, but all these can be found elsewhere. Here alone we find a truly homœopathic institution, with the vital essence of the science (apart from matter!) infused, as it were, into the very bricks and mortar of the place. Here it breathes and has its being unsullied by weak-kneed compromise with any and every other kind of practice, custom, or habit in medicine.

The cause of our rejoicing should, therefore, lie in the spirit which actuated the enterprise, and the brave and stubborn maintenance of principle on the part of its founders.

At the laying of the corner-stone of this edifice, three years ago, Dr. Guernsey used these memorable words:

"I am well aware the word Homœopathy has become a cloak for a multitude of evils in the practice of medicine; that it is made to comprehend much that is spurious and entirely foreign to the real spirit and successful operation of the system. But I hope, in this instance, it will prove an exception; that the edifice about to rise from this corner-stone, in its grand proportions, will be used to exemplify and perpetuate that purity and simplicity in things medical which were intended by its projectors; that neither eclecticism nor any other ism shall be allowed to tarnish its pure walls or impair the usefulness of the institution."

Dr. Guernsey's warnings have been regarded, and, thanks to the brave women of the Executive Board, his hopes for the institution have been realized. He recognized the dangers which might befall this undertaking, not from the antagonism of the allopathic physicians, but from the virulent enmity of that class of physicians who arrogate to themselves the name of homœopath, but practice what they please.

The founders of this hospital were thoroughly impressed with the truths and eminent results in the practice of a few physicians who adhered strictly to a law in therapeutics, who never deviated from its conscientious application in disease, who found no exigency too great for its beneficial use. This practice these women were determined to extend to those who appealed for assistance to them under this roof.

In their capacity of the Executive Board of a truly homœopathic hospital, they were naturally called upon to be judges also of what constituted Homœopathy and of that which was antagonistic with its tenets. They accordingly set about the task of acquainting themselves with the works of Hahnemann. The *Organon* was as thoroughly studied as their Bible, and the great discoveries incorporated in the *Chronic Diseases* were revealed to them.

This knowledge of the fundamental teachings of Hahnemann's system of medicine saved this hospital from the disgrace of becoming another of the many eclectic establishments which go current under the name of "homœopathic." When Carbolic acid, Quinine pills, compounds of Belladonna, Strychnia, and Hyoscyamus, crude Ergot, Monsell's solution, etc., were called for by the staff of physicians, *this Executive Board of women were honest*, and declined to furnish them, as the use of such agents and compounds is in direct violation of the principles which govern its medical declaration. The staff of physicians resigned, taking the ground that they were to be the judges of what constituted homœopathic practice, and demanded freedom from interference by the Executive Board in their *prerogatives*.

This demand for liberty (or, better, license) on the part of the staff sounds very well, but the argument will not hold water where principle, method, and law are to govern. Suppose, to use a rather rough simile, a watchman is placed in charge of a clock factory, and one of the employees thinks it his *prerogative* to take a sledge-hammer and smash two or three clocks intrusted to the watchman's care, under the pretense that, being employed to make and repair clocks, he has, nevertheless, occasional fancies that the striking of certain clocks is distasteful to him, and when collared by the watchman argues that his *prerogatives* are infringed upon by being interfered with by an ignorant watchman, who knows nothing whatever about making and repairing clocks; I merely ask, who has the right of it, the clockmaker or the watchman who has collared him?

A medical journal, in commenting on the action taken by these ladies, said: "The Board, being *presumably* ignorant of what constitutes Homœopathy, may be excused." Here is just where the editor of this journal shows his ignorance of Homœopathy, as well as his ignorance of the competency of these ladies to judge in this matter. If this editor had taken the trouble to ask the Board their reasons for discharging the incompetent doctors, he would have "presumably" ascertained some very good homœopathic reasons. In their knowledge of the philosophy and institutes of Homœopathy these ladies were the peers of the departing doctors, and it was their solemn duty to interfere with practices which had nothing in common with Homœopathy.

It was our privilege to meet some of the ladies last summer, and we can assure the editor of this journal that we found nothing "presumably" ignorant in them of what constitutes Homœopathy, and, strange as it may seem, they were in other respects quite intelligent. I will venture to add that if the philosophy of our science was as well understood by the majority of professors of materia medica and practice in our so-called homœopathic colleges, fewer eclectics would issue as graduates from their halls.

Why, ladies and gentlemen, I have ocular evidences in my desk at home of the practice of graduates from homœopathic colleges which would make the best doser among the regulars shudder, and if the prescribing doctor had himself taken the doses he gave the poor patient, he would have been a subject for hypodermic injections by the professor who taught him such practice. "Carelessness on the part of the practitioner"—yes, indeed, criminal carelessness—but who is chiefly to blame? *the teacher.*

These ladies distinguished between the pure and spurious. They learned to recognize that which is *new* in the science of medicine *discovered* and elaborated, and not *invented*, by Samuel Hahnemann.

By their researches in homœopathic literature they discovered that only medicines which had been proved upon the healthy were admissible weapons against disease under our law of cure, and that the *characteristics* so derived were to indicate their use in a case which presented similar peculiarities and marked features. They learned that Hahnemann was the first to insist upon the most minute and careful study of the symptoms of a case, discriminating especially between the peculiar and personal indications and those which were common to the disease under consideration.

They learned that *one*, and only one, remedy should be given, and that this remedy should be allowed to act without repetition till its curative action is exhausted.

They learned that substances which were inert in their crude state were changed into useful curative agents by Hahnemann's method of potentizing, and that the most virulent poisons were converted into mild, harmless, but potent curative factors.

They learned that all local treatment must be harmful, as no such thing exists as a local disease.

They, finally, learned that the system of medicine which they cherished, and were determined to put to practical use and test, had nothing whatever to do with nosology (the classification of diseases) as giving an indication for the use of a homœopathic remedy, but that its successful application consisted in strictly individualizing every case, without regard to the name which had been given it.

They were true to their trust, and their convictions were strong enough to keep their work free from defilement.

After courageously ridding the institution of a set of unworthy physicians, a system of persecution was inaugurated against the Executive Board which forced the members of this private Institution to vindicate their position in the public press of Philadelphia, in which they had been attacked by the County Medical Society, before which the departing staff had brought their grievances.

It seems that in Philadelphia Homœopathy has nothing more to fear from allopathic antagonists, but much from that class of physicians calling themselves by the honorable name of homœopathist, but practicing without those laws and methods which are the essence of the art.

Persecutions are perhaps the surest and shortest road to success, provided the persecuted know that they have right on their side and courage enough to defend it. Not only in the science of medicine, but in almost every human undertaking upon which progressive thought and investigation has been brought to bear, have the originators and their adherents been remorselessly persecuted. As an instance, we need only to recall the history of our Hahnemann, who from 1799 to 1821 was obliged, by the malevolence of his colleagues, to change his abode a dozen times. The city of Leipsic, the scientific capital of his country, to which by youthful association he was drawn, and where he expected to find kindly recognition for his valuable labors, treated him so outrageously that he was obliged to flee. Eight years after his death, however, a beautiful bronze statue of the great physician, discoverer, and reformer was unveiled in that city before the same Council which had chased him from the town like a criminal.

The worst enemies of this Institution are, as Dr. Dunham very forcibly said on another occasion, "those who, for the sake of what they call *peace* and *union*, would join hands with what they know to be false! Aye, even though to do it they should have to 'cut off the fanatics' who adhere strictly to Hahnemann—to leave the 'brains' of their system 'out in the cold.'"

I have been told on good authority that Hahnemann gave this answer to a gentleman who visited him in Paris and congratulated the old hero upon having lived to see his system practiced in every country of the globe and upon being able to count his followers by thousands: "Those who call themselves my followers," said Hahnemann, "may perhaps be counted by thousands; those whom I acknowledge can be counted on the fingers of both hands. It has sometimes seemed to me that my discoveries were made half a century too early."

I think we may safely claim that a new era for Homœopathy is dawning, and it is not yet fifty years since Hahnemann made this statement. There are at least five hundred men and women now in the United States alone who are practicing Homœopathy strictly in accordance with the fundamental precept of its author, and who recognize his philosophy of disease and its cure. These men and women will be gradually united with a medical Society already in vigorous existence, not vigorous as yet by its numbers, but strong in its convictions and enthusiastic in the work of further developing the great art of Homœopathy.

While we have women at the head of this Institution such as constitute its present Executive Board we need have no fear that compromises will be made with any practice not strictly in accordance with Hahnemann's methods. The following words of their report to the International Hahnemannian Association is pledge sufficient of their intent and purpose:

"Thus the initiative was taken with a deep sense of the significance of the position assumed, and the two prominent governing ideas were the wise, tender care and sheltering of the unfortunate, and the alleviation of human suffering by the application to diseased conditions of the beneficent principles of Homœopathy '*pure and simple.*' This work was not undertaken lightly or blindly, though some among us did not in the beginning comprehend all that was involved in the step, and many of the obstacles and difficulties have been both a pain and a surprise, but with the labor has come a growing appreciation of the noble science in whose cause the best thought and strength is given, and this should carry its own instant compensation for every perplexity. We are fully aware that truth is all-powerful, yet before it is established its birth in human minds is through exceeding great travail, and only by contrast with its opposite, error, does it become manifest in clearness and perfect purity."

Certainly, for such words and such works we have true cause for rejoicing.

Rev. Dr. Wayland then stepped forward, and after a brief introductory read the dedication hymn that was sung at the laying of the corner-stone of the Sister Dora Hospital, at Walsall, England. The beautiful lines, beginning, "Accept this building, gracious God," were then sung by a choir of ladies.

The venerable Dr. P. P. Wells, of Brooklyn, being present, was called upon for an impromptu address.

The old gentleman arose, and demanded if this was the usual practice of Philadelphia people, to put an old man in such a tight place. He then declared that the mention of the name of the late Dr. Hering brought tears to his eyes. After eulogizing Dr. Hering, Dr. Wells gave an interesting account of his own conversion to Homœopathy. He declared that when he was a physician of the regular school, his object was to direct his medical methods to produce the least mischief. When he had learned Homœopathy, he changed his policy to attempt the greatest benefit. He confessed that there never was a man who said more foolish things against Homœopathy than he did before he understood it. But when he undertook experiments to disprove it, he became converted. These very experiments were such a revelation that he was confounded, and his hostility to the new system removed. After that he never gave but two doses of old school physic, and the two patients who got these doses were nearly killed by them, and the Doctor was glad of it.

What is it that makes Homœopathy? It is neither the much or little. The quantity has nothing to do with it. It is a *law* of cure. It is the invention of Almighty God; it is a revolution which He made through the illustrious Samuel Hahnemann. Such a principle, coming from such an Author, should be profoundly respected.

But this is a pretty poor kind of world for truth to make progress. If the world is not disposed to examine a truth and accept it fully, it accepts so much as is convenient, and throws away the rest. The doctors who so suddenly resigned from this hospital were of this order of the world. Why was it that they resigned? It was because they *don't know* the principle they profess. They don't know. They have never been taught. There is no institution in this country save one where Homœopathy is actually taught. In New York is a so-called homœopathic institution which openly declares that it does not pretend to teach Homœopathy.

Confidence in this homœopathic principle of treatment will come if one has confidence in Almighty God that He will take care of His own.

Addresses followed by Judge W. N. Ashman, of the Orphans' Court, and Miss A. E. Ramborger, of the Executive Board. The latter was a thoroughly prepared statement of the financial condition of the hospital, its history, etc., and ended in an eloquent appeal for funds to clear away the debt of thirty-five thousand dollars that yet remains upon the buildings.

(Extracts from this statement will be published in subsequent numbers of this journal.)

The ceremonies closed with the doxology and benediction.

---

